# Stress perception, academic motivation, and mental well-being: evidence from Chinese undergraduate students

**DOI:** 10.3389/fpsyg.2026.1789134

**Published:** 2026-03-16

**Authors:** Shanshan Hao, Xiaoshu Xu, Xibing Wang, Guangzhao Chai, Wei Wei

**Affiliations:** 1 Faculty of Applied Sciences, Macao Polytechnic University, Macao, Macao SAR, China; 2 School of Foreign Studies, Wenzhou University, Zhejiang, China; 3 Mechanical and Electrical Vocational and Technical College, Kunming, Yunnan, China

**Keywords:** stress perception, academic motivation, mental well-being, educational psychology, psychological adaptation, digital learning environment, learning technology, university students

## Abstract

**Objective:**

Stress is a pervasive feature of university students’ learning experiences, yet its psychological consequences depend less on stress exposure than on how stress is perceived and cognitively appraised. Drawing on the transactional model of stress and Self-Determination Theory, this study examines the relationships among stress perception, academic motivation, and mental well-being, with a focus on the mediating role of academic motivation.

**Methods:**

A sample of 322 Chinese undergraduate students completed validated self-report measures, including the Perceived Stress Scale, the Short Academic Motivation Scale, and the Short Warwick-Edinburgh Mental Well-being Scale. Data were analyzed using Partial Least Squares Structural Equation Modeling (PLS-SEM).

**Results:**

Perceived stress was positively associated with academic motivation and mental well-being. Academic motivation statistically mediated the association between stress perception and mental well-being.

**Conclusion:**

The findings suggest that the associations between perceived stress, motivation, and mental well-being may be more nuanced than traditionally assumed. Rather than reflecting exclusively maladaptive strain, perceived stress may capture appraisal-based responses linked with academic engagement. These results highlight the importance of cognitive appraisal and motivational processes in understanding student psychological functioning.

## 1 Introduction

The mental well-being of university students has become a prominent concern across higher education systems worldwide. In China, recent national survey evidence suggests that approximately 18.9% of university students report notable mental health symptoms, with anxiety and depressive experiences being particularly prevalent (Yu, 2022). During the university years, academic demands, such as examinations, performance evaluation, and uncertainty about future career prospects, are widely recognized as major sources of stress. As academic environments become increasingly competitive, clarifying how students psychologically adapt to academic stress has become an urgent research priority (Benítez-Agudelo et al., 2025; Karyotaki et al., 2020).

Importantly, the psychological relationship of academic stress cannot be explained by the presence or intensity of stressors alone. A growing body of work indicates that students’ subjective appraisal of stress, commonly conceptualized as stress perception, may be more consequential for motivational and well-being outcomes than objective academic demands (Kennett et al., 2021; Pérez-Jorge et al., 2025). Stress perception reflects the extent to which individuals evaluate situations as unpredictable, uncontrollable, or overwhelming, as well as their perceived capacity to cope with these demands (Cohen et al., 1983; Cohen and Janicki-Deverts, 2012). From the transactional perspective, such cognitive appraisals shape emotional responses, behavioral engagement, and subsequent psychological adjustment (Folkman, 1984, 2020). In academic contexts, maladaptive stress appraisal has been associated with outcomes such as emotional exhaustion, disengagement, procrastination, and poorer mental health (Pike Moore et al., 2025).

At the same time, stress perception does not necessarily operate as a uniformly harmful relations (Steiner-Hofbauer and Holzinger, 2020). Research on eustress suggests that moderate levels of stress, when appraised as manageable, meaningful, or goal-relevant, may enhance alertness, task engagement, and academic performance (Li et al., 2023). Students who interpret academic challenges as opportunities for growth rather than threats may be more likely to sustain engagement and psychological resilience (Wijaya et al., 2025). This dual possibility highlights stress perception as a psychologically dynamic construct: its consequences depend on how academic demands are cognitively evaluated, rather than on demands alone (Freire et al., 2016; Ruiz-Camacho and Gozalo, 2025). From a motivational standpoint, Self-Determination Theory (SDT) emphasizes that motivation is supported when autonomy, competence, and relatedness needs are satisfied; stress appraisals that undermine these needs (particularly perceived competence) may contribute to motivational decline and emotional distress, whereas appraisals aligned with personal goals and perceived control may help preserve motivation (Ahmed and Al Salim, 2024; Metzner et al., 2025).

Empirical evidence further supports a close linkage between academic motivation and mental well-being (Ryzin et al., 2018). Students with higher levels of intrinsic or autonomous motivation more consistently report greater life satisfaction, emotional balance, and resilience, whereas motivational deficits are associated with disengagement, academic avoidance, and elevated stress (Bureau et al., 2022; Vansteenkiste et al., 2020). In parallel, emerging studies increasingly identify motivation-related variables, such as autonomous motivation and self-efficacy, as key mechanisms that may transmit the association of stress perception to mental health outcomes (Mehta et al., 2024; Schönfeld et al., 2016). Taken together, this line of research suggests that academic motivation may function as a psychological conduit through which stress perception is translated into differences in mental well-being.

Despite growing attention to stress, motivation, and well-being, several gaps remain. First, much of the existing literature emphasizes the detrimental consequences of stress, with comparatively less attention to the potentially adaptive role of positively appraised academic stress (Rudland et al., 2020; Yeager et al., 2022). Second, although motivation is frequently proposed as a mediating mechanism, studies that simultaneously examine stress perception, academic motivation, and mental well-being within a unified structural model remain relatively scarce (Rahe and Jansen, 2022). Third, evidence from the Chinese higher education context is still limited, despite cultural norms that place strong emphasis on academic achievement and social evaluation, conditions that may shape both stress experiences and motivational dynamics (Wang et al., 2024; Yu et al., 2018).

To address these gaps, the present study investigates the interrelationships among stress perception, academic motivation, and mental well-being among Chinese undergraduate students. Specifically, the study examines (a) whether stress perception is associated with academic motivation and mental well-being, and (b) whether academic motivation mediates the relationship between stress perception and mental well-being. Using Partial Least Squares Structural Equation Modeling (PLS-SEM) with survey data from Chinese undergraduates, this research aims to clarify how students’ cognitive appraisals of academic stress relate to motivational engagement and psychological well-being. By focusing on stress perception rather than objective stressors, the study seeks to advance a more nuanced understanding of student functioning in high-pressure academic environments and to offer evidence relevant to educational practice and student mental health interventions.

## 2 Literature review

### 2.1 Stress perception and academic motivation

Stress perception refers to individuals’ subjective appraisal of the extent to which environmental demands are experienced as unpredictable, uncontrollable, or overwhelming relative to available coping resources (Cohen et al., 1983). In academic settings, stress perception captures how students cognitively interpret stressors such as examinations, workload, and performance evaluation, rather than the objective presence of those demands (Jamieson et al., 2022). Accumulating evidence suggests that stress perception is a more proximal predictor of students’ motivation related and psychological outcomes than objective exposure to academic stressors (Cabras et al., 2024).

The transactional model of stress further explains why appraisal is central to motivational functioning (Bartels et al., 2023). Specifically, cognitive appraisal processes shape emotional responses and behavioral engagement under stress (Folkman, 1984; Yeo and Ong, 2024). When academic demands are appraised as threatening or uncontrollable, students are more likely to report reduced self-efficacy, emotional exhaustion, and disengagement from learning activities (Islam et al., 2022). Empirically, higher levels of perceived stress have been consistently associated with lower academic motivation, particularly intrinsic motivation, as perceived stress may erode students’ sense of autonomy and competence (Nguyen and Chen, 2023).

However, the motivational implications of stress perception are not uniformly negative. Research on eustress indicates that moderate stress, when appraised as manageable or growth-promoting, can enhance motivation by increasing alertness, task involvement, and commitment to goals (Shirish et al., 2023). Students who interpret academic challenges as opportunities for development rather than threats may be more likely to sustain engagement and persist in goal-directed behavior (Skinner et al., 2016; Wijaya et al., 2025). This dual role underscores the dynamic nature of stress perception and suggests that motivational outcomes depend largely on appraisal patterns rather than stress exposure alone (Folkman, 2020; Riepenhausen et al., 2022).

Despite growing interest in stress and motivation, much of the empirical literature continues to emphasize the detrimental motivational consequences of stress, with comparatively limited attention to potentially adaptive functions in higher education contexts. In addition, evidence from non-Western educational settings remains relatively scarce. These gaps highlight the need for further investigation of how stress perception relates to academic motivation among university students, particularly in culturally demanding academic environments such as China.

### 2.2 Stress perception and mental well-being

Mental well-being encompasses positive psychological functioning, including emotional balance, life satisfaction, and a sense of purpose and control (Pakpour et al., 2024). Within academic contexts, students’ mental well-being is closely tied to how they perceive and respond to academic demands. A growing body of research indicates that perceived stress is a robust predictor of psychological distress, anxiety, and depressive symptoms among university students (Ansari et al., 2025; Zhang and Li, 2026).

Cognitive appraisal theory provides a basis for understanding this linkage. When students appraise academic demands as exceeding their coping capacity, stress responses are more likely to co-occur with negative affect, emotional exhaustion, and diminished psychological functioning (Malykhin et al., 2025; Rnic et al., 2023). Conversely, adaptive appraisal processes, such as perceiving stress as manageable or meaningful, are associated with more effective emotion regulation and greater psychological resilience (Marciniak et al., 2024). Accordingly, stress perception is not merely a reflection of academic pressure; it is a psychological lens that shapes how academic experiences are emotionally and cognitively processed.

Empirical evidence also indicates that stress perception may exert both immediate and sustained association on mental well-being. Cross sectional and longitudinal studies show that higher perceived stress is associated with lower life satisfaction and poorer emotional stability, whereas adaptive appraisal can buffer the negative psychological consequences of academic pressure (Demichelis et al., 2024). These findings reinforce the view that students’ mental well-being is shaped not only by academic demands per se, but more critically by how those demands are cognitively interpreted.

### 2.3 Academic motivation and mental well-being

Academic motivation is a central determinant of students’ engagement, persistence, and psychological functioning. Self-Determination Theory (SDT) distinguishes autonomous motivation, such as intrinsic motivation and identified regulation, from controlled motivation driven by external pressure or avoidance (Ryan and Deci, 2000). Autonomous motivation is consistently linked to adaptive academic and psychological outcomes, including greater life satisfaction, emotional balance, and psychological resilience.

A substantial body of evidence demonstrates a positive association between academic motivation and mental well-being (Taylor et al., 2014; Grassinger et al., 2024). Intrinsically motivated students tend to report higher positive affect, lower emotional exhaustion, and greater satisfaction with academic life (Karimi and Sotoodeh, 2020). In contrast, low motivation and a motivation are associated with academic disengagement, procrastination, and elevated psychological distress (Oláh et al., 2023). Taken together, these findings suggest that academic motivation can function as a protective psychological resource, especially in high-pressure learning environments.

Although the motivation and well-being relationship can be bidirectional, stress related models commonly conceptualize academic motivation as a mechanism through which environmental and psychological conditions relate to mental well-being. Motivated students are more likely to maintain a sense of purpose, perceive competence, and regulate emotions effectively, which collectively supports sustained psychological functioning.

### 2.4 The mediating role of academic motivation

Integrating these strands of research, an increasing number of studies suggest that academic motivation may mediate the relationship between stress perception and mental well-being (Jiang and Tanaka, 2022; Passeggia et al., 2023). High levels of perceived stress are associated with diminished motivation, which in turn contributes to emotional exhaustion and reduced psychological well-being (Freire et al., 2016; Ruiz-Camacho et al., 2025). Meta-analytic and longitudinal evidence indicates that motivation-related variables, such as academic self-efficacy and autonomous motivation, account for a substantial proportion of the indirect association of stress on mental health outcomes (Zhang et al., 2022; Liu et al., 2024).

From an SDT perspective, stress perception may weaken motivation by disrupting the satisfaction of basic psychological needs, particularly competence and autonomy (Ryan and Deci, 2020, 2000; Vansteenkiste et al., 2020). When students appraise academic demands as overwhelming or uncontrollable, they may withdraw effort and reduce engagement, thereby lowering motivation and increasing vulnerability to declines in mental well-being (Kennett et al., 2021; Spătaru et al., 2024). Conversely, when stress is appraised as manageable, motivation may be preserved or strengthened, which can buffer the adverse psychological association of academic pressure (Jamieson et al., 2022; Petri-Romão et al., 2025).

Despite strong theoretical rationale for this mediation pathway, relatively few studies have examined stress perception, academic motivation, and mental well-being simultaneously within a unified structural model, particularly among Chinese undergraduate populations. Addressing this gap requires an integrative empirical approach capable of estimating both the direct association of stress perception on mental well-being and the indirect association transmitted through academic motivation.

## 3 Methods

This study employed a standardized questionnaire design to examine the interrelationships among stress perception, academic motivation, and mental well-being among Chinese undergraduate students. Survey methodology enabled the efficient collection of large-scale self-reported data suitable for latentvariable modeling and the estimation of both direct and indirect pathways linking perceived stress to motivational processes and psychological well-being in higher education.

### 3.1 Sample and sampling

Participants were full-time undergraduates enrolled in traditional degree programs at comprehensive universities in Mainland China. To ensure comparability in academic expectations and learning conditions, students from vocational or other non-traditional pathways were excluded. University undergraduates were selected because they are consistently exposed to academic evaluation demands and routinely engage with learning technologies that intersect with stress, motivation, and well-being processes (Getenet et al., 2024).

The final sample size (*n* = 322) exceeded commonly recommended thresholds for PLS-SEM estimation and provided adequate statistical power. Of the respondents, 30.5% identified as male and 69.5% as female. Regarding age, 71.78% were 18–20 years old, 25.58% were 21–24, and 2.64% were under 18. This demographic distribution is broadly consistent with the typical profile of undergraduate cohorts in Mainland China, supporting the interpretability of findings within this educational context. While convenience sampling may limit representativeness, this approach is commonly adopted in student-based behavioral research.

### 3.2 Research instruments

A structured questionnaire comprising three validated psychometric scales was used to assess stress perception, academic motivation, and mental well-being. Instruments were selected for their established reliability, construct validity, and applicability to university populations experiencing academic pressure and developmental transitions.

Stress perception was measured using the 14-item Perceived Stress Scale (PSS) developed by Cohen et al. (1983). The PSS captures perceived helplessness and perceived self-efficacy and assesses the degree to which situations are experienced as unpredictable, uncontrollable, and overwhelming over the past month. A sample item is: “In the last month, how often have you felt that you were unable to control the important things in your life?” Items were rated on a 6-point Likert scale from 0 (“Never”) to 5 (“Very often”), with higher summed scores indicating greater stress perception. Internal consistency estimates indicated satisfactory reliability (Cronbach’s *α* = 0.903). Missing data levels were minimal (less than 5% per indicator). Missing values were handled using single mean imputation, which is considered acceptable when missingness is low. For descriptive purposes only, stress scores were categorized using tertile cut-points.

Academic motivation was assessed using the 14-item Short Academic Motivation Scale (SAMS) developed by Kotera et al. (2023). The SAMS measures intrinsic motivation, extrinsic motivation, and amotivation across seven subscales and has demonstrated robust psychometric properties for large scale research. A representative item is: “I study because I enjoy learning new things.” Responses were recorded on a 7-point Likert scale from 1 (“Completely disagree”) to 7 (“Strongly agree”), with higher scores reflecting stronger academic motivation. Internal consistency estimates indicated satisfactory reliability (Cronbach’s α = 0.929).

Mental well-being was measured using the Short Warwick–Edinburgh Mental Well-being Scale (SWEMWBS), which emphasizes positive psychological functioning (e.g., emotional balance, interpersonal functioning, and perceived purpose and control) rather than the absence of symptoms (Pakpour et al., 2024). A sample item is: “I’ve been feeling optimistic about the future.” Items were rated on a 5-point Likert scale from 1 (“None of the time”) to 5 (“All of the time”), with higher total scores indicating greater mental well-being. Internal consistency estimates indicated satisfactory reliability (Cronbach’s α = 0.966).

### 3.3 Data collection

Convenience sampling was used to recruit participants. The questionnaire was administered through two channels: (a) in-person distribution in randomly selected undergraduate classes and (b) online dissemination via the Wenjuanxing (Sojump) platform. In total, 420 questionnaires were distributed and 387 were returned. Following data screening and quality control, including the removal of incomplete responses, multivariate outliers, and unengaged response patterns (e.g., straight-lining), 322 valid cases were retained for analysis, yielding an effective response rate of 83.9%. The final sample size falls within commonly cited recommendations for structural equation modeling in behavioral research (30–500).

### 3.4 Data analysis

Prior to model estimation, a structured preprocessing protocol was implemented. First, duplicate submissions were removed. Second, responses with excessive missingness or logical inconsistencies were excluded. Third, categorical variables (e.g., gender) were standardized and continuous variables (e.g., age) were formatted consistently. Fourth, outliers were screened using distributional diagnostics. No extreme cases requiring exclusion were identified (Côté et al., 2024).

Missing values were handled using single mean imputation. This approach was considered appropriate given the low proportion of missingness and the robustness of PLS-SEM to minor data imperfections. Moreover, imputation was performed prior to latent variable estimation, minimizing potential estimation bias. The hypothesized model was tested using Partial Least Squares Structural Equation Modeling (PLS-SEM). PLS-SEM was selected due to its suitability for prediction-oriented modeling and robustness to distributional assumptions. PLS-SEM was selected because it is robust to non-normality, well-suited for estimating models with multiple latent constructs and indirect association, and emphasizes prediction oriented evaluation features often aligned with applied behavioral research (Sarstedt and Liu, 2024). Analyses were conducted in SmartPLS 4. The measurement model was evaluated prior to hypothesis testing, including indicator performance and internal consistency, convergent validity (e.g., AVE), and model-based estimates of structural relations (path coefficients). The structural model evaluation focused on estimating direct and indirect association linking stress perception, academic motivation, and mental well-being, along with overall explanatory performance.

### 3.5 Ethical consideration

This study complied with internationally recognized ethical standards and received approval from the corresponding author’s institution (#WZU2025-0203F). Participants were informed of the study purpose, procedures, and data protection measures prior to participation. Informed consent was obtained electronically or in writing, participation was voluntary, and respondents could withdraw at any time without penalty. For participants under 18 years old, parental or legal guardian consent was also obtained. No personally identifiable information was collected; responses were anonymized and stored securely with access restricted to the research team. Procedures followed the ethical principles of the Declaration of Helsinki and aligned with relevant data protection requirements, including the General Data Protection Regulation (GDPR).

## 4 Results

### 4.1 Measurement model

To evaluate the hypothesized relationships among stress perception, academic motivation, and mental well-being, the measurement and structural models were estimated using Partial Least Squares Structural Equation Modeling (PLS-SEM) in SmartPLS 4. PLS-SEM is appropriate for models with multiple latent variables, moderate sample sizes, and potential departures from multivariate normality (Sarstedt and Liu, 2024). Although PLS-SEM does not require multivariate normality, Mardia’s test was conducted to diagnose multivariate skewness and kurtosis. To further strengthen statistical inference, nonparametric bootstrapping with 5,000 resamples was applied. Missing data levels were minimal (less than 5% per indicator). Missing values were handled using single mean imputation, which is considered acceptable when missingness is low. The final sample size (*n* = 322) exceeded recommended thresholds for PLS-SEM estimation.

#### 4.1.1 Reliability and convergent validity

Standardized indicator loadings met recommended criteria. All retained indicators loaded above 0.50, with most exceeding 0.80, supporting item reliability. Loadings ranged from 0.752 to 0.877 for Stress Perception (SP), 0.731 to 0.908 for Academic Motivation (AM), and 0.609 to 0.909 for Mental Well-Being (MWB) (see Table 1).

**Table 1:** Assessment of lower order components.

Construct	Items	Loading	α	CR	AVE
Stress perception (SP)	SP1	0.877	0.903	0.909	0.674
	SP2	0.838			
	SP3	0.837			
	SP4	0.807			
	SP5	0.752			
	SP6	0.813			
Academic motivation (AM)	AM1	0.863	0.929	0.952	0.608
	AM2	0.883			
	AM3	0.908			
	AM4	0.819			
	AM5	0.785			
	AM6	0.850			
	AM7	0.841			
	AM8	0.891			
	AM9	0.855			
	AM10	0.820			
	AM11	0.731			
Mental well-being (MWB)	MWB1	0.800	0.966	0.970	0.699
	MWB2	0.829			
	MWB3	0.783			
	MWB4	0.609			
	MWB5	0.827			
	MWB6	0.875			
	MWB7	0.879			
	MWB8	0.909			
	MWB9	0.855			
	MWB10	0.903			
	MWB11	0.872			
	MWB12	0.767			
	MWB13	0.851			
	MWB14	0.894			

Internal consistency estimates were assessed using Cronbach’s alpha (*α*) and composite reliability (CR). As shown in Table 1, α and CR values exceeded the conventional 0.70 benchmark across constructs, indicating satisfactory reliability consistency.

Convergent validity was supported by AVE values above 0.50 for all constructs Table 1, indicating that each latent construct explained a substantial proportion of variance in its indicators relative to measurement error.

#### 4.1.2 Discriminant validity

Discriminant validity was evaluated using the Fornell–Larcker criterion. The square root of each construct’s AVE (diagonal) exceeded its correlations with other constructs Table 2, indicating that SP, AM, and MWB were empirically distinguishable. These results indicate that the constructs were empirically distinct.

**Table 2:** The Fornell-Larcker discriminant validity.

Construct	SP	AM	MW
Stress perception	0.821		
Academic motivation	0.301	0.780	
Mental well-being	0.686	0.485	0.836

Discriminant validity was assessed using the Heterotrait–monotrait ratio (HTMT). As shown in Table 3, all HTMT values were below the conservative threshold of 0.85, indicating satisfactory discriminant validity among the constructs. Specifically, the HTMT values ranged from 0.330 to 0.725, suggesting that the constructs are empirically distinct while still demonstrating theoretically meaningful associations.

**Table 3:** Heterotrait–monotrait ratio (HTMT) matrix.

Construct	HTMT
SP-AM	0.330
MWB-AM	0.511
MWB-SP	0.725

#### 4.1.3 Descriptive statistics and correlation analysis

To enhance transparency and provide preliminary insight into the relationships among the constructs, descriptive statistics and Pearson correlation coefficients are reported in Table 4. The results indicate that stress perception, academic motivation, and mental well-being were significantly intercorrelated, providing preliminary support for the structural model estimation. This pattern is consistent with appraisal-based interpretations of perceived stress, although the present study did not directly distinguish between challenge and threat appraisals.

**Table 4:** Descriptive statistics and correlations.

Construct	Mean	SD	SP	AM	MWB
Stress perception	41.527	6.231	1		
Academic motivation	43.876	10.911	0.301	1	
Mental well-being	61.938	14.085	0.686	0.485	1

No excessively high correlations were observed (r < 0.85), indicating no multicollinearity concerns.

Descriptive statistics (means and SDs) and Pearson correlations among stress perception, academic motivation, and mental well-being are presented in Table 4 (e.g., SP–MWB r = 0.686; SP–AM r = 0.301; AM–MWB r = 0.485). All constructs were moderately correlated, providing preliminary support for the proposed relationships while indicating no severe multicollinearity.

#### 4.1.4 Common method bias assessment

Given that all constructs were measured using self-report instruments at a single time point, common method variance (CMV) was assessed. Following Kock (2015), full collinearity variance inflation factors (VIFs) were examined. All VIF values were below the conservative threshold of 3.3, indicating that common method bias is unlikely to substantially affect the results.

### 4.2 Structural model assessment

Before estimating the full structural model, Pearson correlation analysis indicated significant positive associations among stress perception (SP), academic motivation (AM), and mental well-being (MWB) (all *p* < 0.01), providing preliminary support for the hypothesized links.

The structural model was then tested using PLS-SEM with bootstrapping (5,000 resamples) to obtain robust standard errors, *t*-values, and *p*-values (Sarstedt and Liu, 2024). Bootstrapping results indicated that all hypothesized paths were statistically significant. Specifically, stress perception was positively associated with academic motivation (*β* = 0.301, t = 4.488, *p* < 0.001) and mental well-being (*β* = 0.594, t = 9.218, *p* < 0.001). Academic motivation was also positively is associated with mental well-being (*β* = 0.306, t = 6.091, *p* < 0.001). Table 5 reports the standardized path coefficients and inferential statistic.

**Table 5:** Path coefficients and significance levels (*N* = 322).

Path	Path coefficient	SD	T-statistics	*P*-values	Hypothesis
SP-AM	0.301	0.067	4.488	0.000	H1 Accepted
SP-MWB	0.594	0.064	9.218	0.000	H2 Accepted
AM-MWB	0.306	0.050	6.091	0.000	H3 Accepted

To evaluate predictive relevance, Stone–Geisser’s Q^2^ was computed via blindfolding. Following Hair et al. (2019), Q^2^ values indicated substantial predictive relevance for the endogenous constructs. As shown in Table 6, Q^2^ values for SP, AM, and MWB all exceeded this threshold, indicating strong out of sample predictive capability of the model (Hair et al., 2019).

**Table 6:** Result of Q^2^ level assessment.

Construct	SSO	SSE	Q^2^ (=1-SSE/SSO)
Stress perception	2,322	1065.678	0.541
Academic motivation	5,418	2408.833	0.555
Mental well-being	5,418	1873.457	0.654

Although the positive association between stress perception and mental well-being may appear counterintuitive given the traditional deficit-oriented view of perceived stress, we clarified that the Perceived Stress Scale reflects subjective cognitive appraisal rather than objective stress exposure. Therefore, the observed association should be interpreted cautiously. This interpretation is consistent with contemporary appraisal-based models of stress, which distinguish between challenge-oriented and threat-oriented perceptions.

### 4.3 Structural model visualization and interpretation

**Figure 1:**
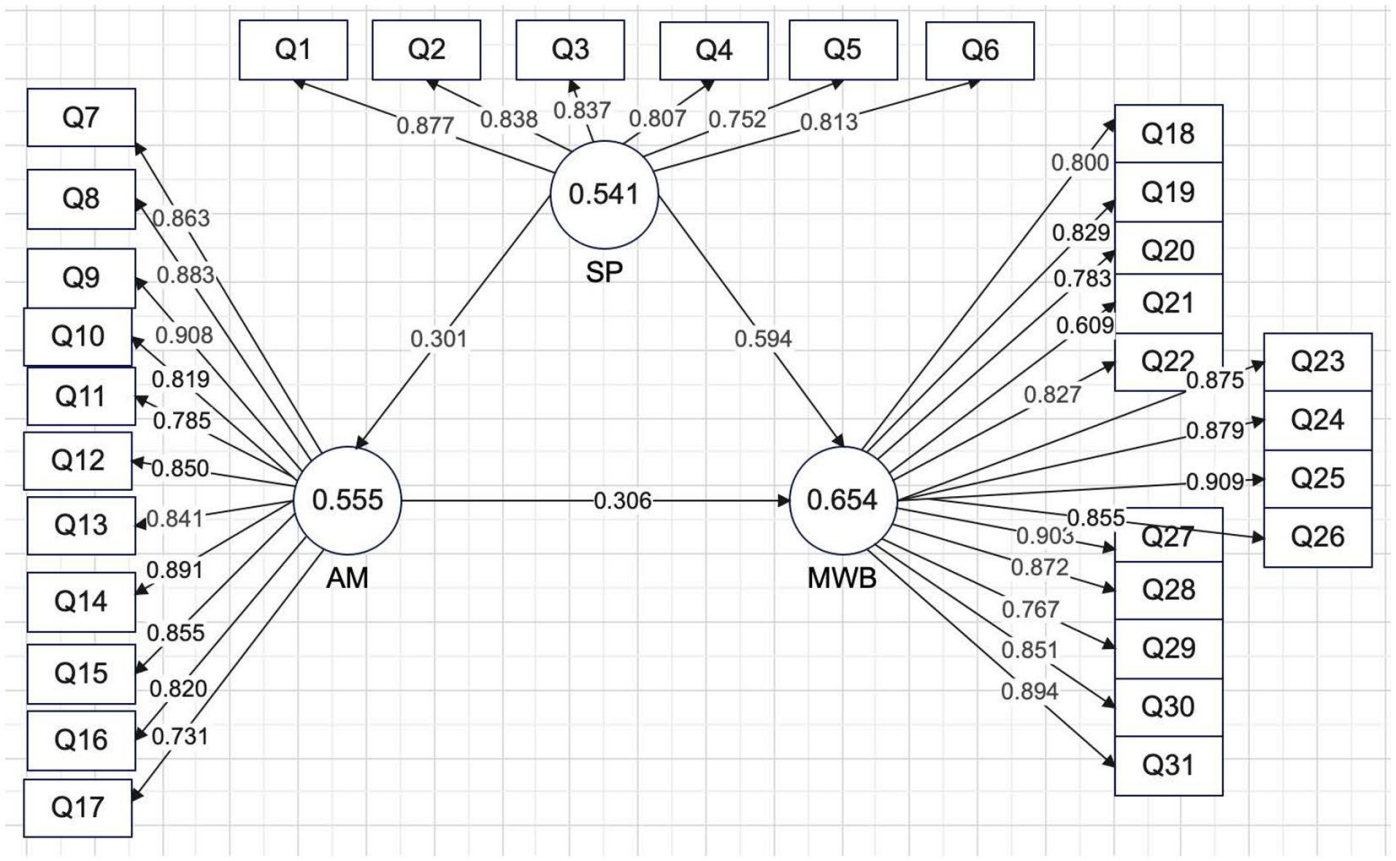
Structural model with path coefficients and significance levels.

Figure 1 depicts the validated structural model, including the direct paths from stress perception (SP) to academic motivation (AM) and mental well-being (MWB), and the path from AM to MWB. Given that both SP → MWB and SP → AM → MWB component paths were significant, the pattern of coefficients is consistent with partial mediation: stress perception is associated with mental well-being both directly and indirectly through academic motivation. In practical terms, the model suggests that stress perception was statistically associated with academic motivation, which in turn was associated with mental well-being, which in turn relates to their reported mental well-being.

### 4.4 Visual summary of the validated model

**Figure 2:**
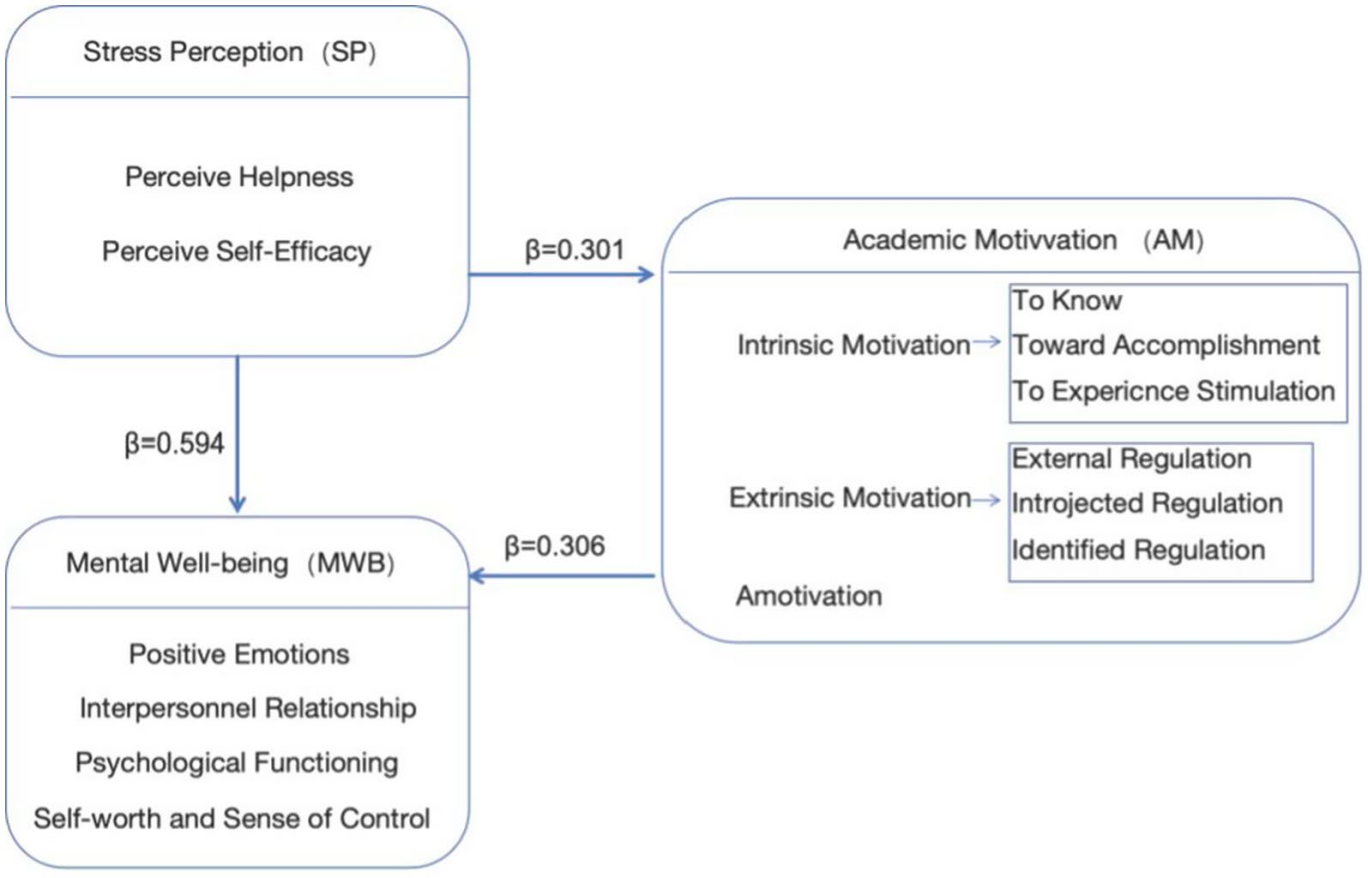
Three-factor statistical verification model.

Taken together, measurement results supported reliable and valid assessment of SP, AM, and MWB, and the structural results supported the hypothesized links with strong predictive relevance (Hair et al., 2019). The final model highlights academic motivation as a meaningful explanatory pathway connecting stress perception to mental well-being, complementing the direct association between stress perception and well-being, see Figure 2.

## 5 Discussion

The present study investigated the interrelationships among stress perception, academic motivation, and mental well-being among Chinese undergraduate students, adopting a cognitive–motivational perspective grounded in the transactional model of stress and Self-Determination Theory (SDT). By foregrounding students’ subjective appraisal of academic demands rather than objective stress exposure, the findings contribute to a more nuanced understanding of how academic stress is psychologically processed and translated into motivational engagement and well-being outcomes (Folkman, 2020, 1984; Ryan and Deci, 2020). Overall, the results indicate that stress perception, academic motivation, and mental well-being are systematically associated rather than independent constructs.

### 5.1 Stress perception as a motivationally relevant psychological signal

One central finding of this study is that stress perception was significantly associated with academic motivation. This association should not be interpreted as evidence that stress is inherently beneficial. Rather, it suggests that perceived stress may function as a psychologically meaningful signal reflecting the salience, importance, and subjective demands of academic tasks. When students appraise academic demands as manageable, meaningful, or aligned with personal goals, perceived stress may coexist with, rather than undermine, motivational engagement (Skinner et al., 2022; Tormon et al., 2023). Importantly, this pattern is unlikely to be attributable to scoring artifacts. Reverse-coded items of the Perceived Stress Scale were handled in accordance with standard scoring procedures, and internal consistency indices indicated satisfactory reliability. The observed direction of association therefore warrants theoretical rather than methodological interpretation.

This interpretation aligns with contemporary distinctions between challenge oriented and threat oriented stress appraisals. Challenge appraisals are typically characterized by perceived controllability, sufficient coping resources, and anticipated gains, whereas threat appraisals involve expectations of harm, loss, or failure (Palmwood and McBride, 2019). Empirical research consistently demonstrates that challenge-oriented appraisals are associated with higher engagement, persistence, and autonomous motivation, while threat appraisals is associated with avoidance behaviors, emotional exhaustion, and disengagement (Bakker, 2024; Glaser and Hecht, 2013). The present findings extend this literature by showing that, at the level of subjective perception, elevated perceived stress does not uniformly correspond to reduced motivational functioning.

From the perspective of the transactional model of stress, this pattern underscores the importance of secondary appraisal, students’ perceived coping capacity and sense of control, in shaping motivational outcomes (Folkman and Moskowitz, 2000). When students believe they possess adequate competence, strategies, and support to meet academic demands, stress is more likely to activate approach oriented coping and sustained effort rather than motivational withdrawal. Longitudinal evidence further suggests that challenge appraisals is associated with increases in engagement over time, whereas threat appraisals is associated with declines (Ewing and Hamza, 2023; Spătaru et al., 2024). Although the present study is cross-sectional, the observed association is consistent with these dynamic accounts.

Within the Chinese higher education context, sociocultural norms emphasizing effort, perseverance, and self-improvement may further shape the motivational meaning of stress. Research grounded in SDT suggests that students in East Asian contexts may be more likely to internalize academic demands and interpret pressure as a normative and morally meaningful component of success (Wang et al., 2022; Yu et al., 2018). In such contexts, stress perception may reflect variations in how students cognitively interpret academic demands rather than exclusively representing maladaptive strain.

### 5.2 Stress perception and mental well-being: moving beyond a deficit oriented view

Stress perception was also significantly associated with mental well-being. Traditionally, perceived stress has been conceptualized as a primary risk factor for psychological distress, anxiety, depression, and burnout (Malykhin et al., 2025; Rnic et al., 2023). However, an emerging body of research emphasizes that the psychological consequences of stress depend critically on appraisal patterns rather than stress intensity alone (Petri-Romão et al., 2025; Riepenhausen et al., 2022). Thus, higher perceived stress scores may capture variations in cognitive appraisal rather than exclusively reflecting maladaptive psychological strain.

In the present study, mental well-being was operationalized as positive psychological functioning, including emotional balance, optimism, and perceived competence (Pakpour et al., 2024). From this perspective, Students who appraise academic demands as manageable may maintain psychological functioning despite elevated stress perception. Perceived stress reflects subjective appraisal processes rather than objective stress exposure. Therefore, the observed association should be interpreted cautiously (Jamieson et al., 2022; Maier and Seligman, 2016).

Empirical studies in higher education contexts increasingly support this dual perspective. Stress reappraisal interventions and challenge oriented stress beliefs have been shown to improve emotional outcomes, academic confidence, and resilience among university students (Bosshard et al., 2025). Moreover, large-scale studies indicate that moderate levels of perceived stress, when accompanied by high perceived control, are associated with better psychological adjustment and lower emotional exhaustion (Amanvermez et al., 2023; Khatri et al., 2024). The present findings extend this literature by providing evidence from a high-pressure, non-Western educational context and underscore the importance of distinguishing adaptive from maladaptive forms of perceived stress when evaluating student well-being.

### 5.3 Academic motivation as a psychological resource for mental well-being

Consistent with SDT, academic motivation was positively is associated with mental well-being. SDT posits that autonomous forms of motivation, such as intrinsic motivation and identified regulation, support well-being by satisfying individuals’ basic psychological needs for autonomy, competence, and relatedness (Ryan and Deci, 2000, 2020). When students experience academic engagement as self-endorsed and personally meaningful, learning activities are more likely to contribute to vitality, purpose, and emotional stability.

A substantial body of empirical research supports this association. Students with higher levels of intrinsic or autonomous motivation tend to engage more deeply with learning tasks, persist during difficulty, and employ adaptive coping strategies, all of which contribute to psychological well-being (Grassinger et al., 2024; Taylor et al., 2014). Conversely, low motivation and amotivation are associated with disengagement, emotional exhaustion, and heightened vulnerability to stress-related difficulties (Bureau et al., 2022; Vansteenkiste et al., 2020). The present findings reinforce the view that academic motivation functions not only as a determinant of learning outcomes but also as a critical psychological resource underpinning mental well-being in demanding educational environments.

### 5.4 Academic motivation as a partial mediator between stress perception and well-being

A key contribution of this study lies in identifying academic motivation as a partial mediator between stress perception and mental well-being. This finding suggests that stress perception relates to well-being through at least two pathways: a direct pathway reflecting the immediate emotional and cognitive burden of perceived stress, and an indirect pathway operating through motivational functioning. When stress is appraised negatively, motivational resources may be depleted, increasing the likelihood of disengagement and diminished well-being. In contrast, when stress is appraised as manageable or meaningful, motivation may be preserved or strengthened, supporting adaptive regulation and engagement (Daniilidou et al., 2025; Schönfeld et al., 2016).

The partial mediation pattern is statistically informative. It indicates that academic motivation accounted for a substantive, but not exhaustive, proportion of the association. This implies that additional mechanisms, such as coping strategies, emotion regulation, and social support, likely operate alongside motivation (Freire et al., 2016; Ruiz-Camacho et al., 2025). Accordingly, future theoretical models would benefit from specifying multiple, parallel pathways to more fully capture the complexity of students’ psychological adaptation to academic stress. Importantly, mediation results derived from cross-sectional data reflect statistical decomposition of associations rather than causal or temporal mechanisms.

### 5.5 Theoretical implications

This study contributes to theory and scholarship in several ways. First, by establishing a significant link between stress perception and academic motivation, the findings extend prior work that has often emphasized stress primarily as a risk factor. Consistent with the broader distinction between challenge and threat appraisals, the results align with the view that stress perception is not inherently maladaptive; its implications depend on how academic demands are cognitively interpreted. This provides further support for a more differentiated conceptualization of stress in higher education, in which perceived stress may shape engagement-related processes rather than simply eroding them.

Second, the results are consistent with Self-Determination Theory (SDT) in highlighting academic motivation as a psychologically meaningful mechanism connecting stress perception to mental well-being. When stress is appraised in ways that undermine basic psychological needs, particularly competence and autonomy, students may experience motivational disruption and, consequently, poorer well-being. Conversely, when stress is experienced as manageable or aligned with personally meaningful goals, motivation may be sustained, thereby supporting well-being. In this sense, the present findings reinforce SDT’s emphasis on motivational quality as a central determinant of adaptive functioning under demanding conditions.

Third, by empirically testing motivation as a mediator, the study advances the literature beyond bivariate stress–well-being associations and toward mechanism-focused explanation. The observed partial mediation is theoretically informative: it indicates that academic motivation explains part, but not all, of the association between stress perception and mental well-being. This implies that additional pathways—such as emotion regulation, coping strategies, or social support—may operate in parallel. Accordingly, future theory-building would benefit from integrative models that specify multiple mediators and allow a more complete characterization of students’ psychological adjustment under academic pressure.

### 5.6 Practical implications

The findings also carry implications for higher education practice. First, the centrality of stress perception suggests that institutions should address not only the magnitude of academic demands but also how students interpret those demands. Pedagogical practices that emphasize mastery-oriented goals, provide formative feedback, and communicate realistic performance expectations may help students appraise academic challenges as manageable rather than overwhelming.

Second, the mediating role of academic motivation indicates that motivation-supportive teaching may function as a well-being intervention. Instructional approaches that support autonomy (e.g., meaningful choice and rationale), strengthen competence (e.g., structured scaffolding and attainable milestones), and cultivate relatedness (e.g., supportive teacher–student interactions) may help buffer the negative well-being correlates of perceived stress by sustaining students’ engagement and sense of purpose.

Third, university stress management initiatives may be strengthened by integrating motivational components. Rather than focusing exclusively on stress reduction—which may be neither feasible nor desirable in achievement-oriented contexts—programs could incorporate appraisal-based strategies (e.g., reframing demands), competence-building supports (e.g., study skills and academic self-efficacy), and goal clarification to help students maintain motivation under pressure. Such combined approaches may be particularly relevant in high-pressure higher education settings, where stress is pervasive and the key practical question is often how to regulate and channel it effectively.

## 6 Conclusion

This study examined the interrelationships among stress perception, academic motivation, and mental well-being among Chinese undergraduate students. By foregrounding students’ subjective appraisal of academic demands rather than objective stress exposure, the findings provide evidence that stress perception is systematically related to both academic motivation and mental well-being. Moreover, academic motivation partially mediated the association between stress perception and mental well-being, underscoring motivation as a meaningful psychological pathway through which stress appraisals are linked to students’ positive psychological functioning.

The results contribute to educational psychology by reinforcing that academic stress should not be conceptualized solely in terms of external demands or as an inherently detrimental condition. Instead, students’ cognitive interpretation of academic demands appears pivotal for shaping motivational engagement and well-being. When academic challenges are appraised as manageable or personally meaningful, students who appraise academic demands as manageable may be more likely to sustain motivation. This conclusion is consistent with contemporary perspectives that emphasize appraisal processes and motivation as central mechanisms of adaptation in learning environments.

Practically, the findings highlight the value of motivation supportive educational practices under conditions of academic pressure. Interventions that strengthen autonomous motivation, support perceived competence, and promote adaptive appraisal may help students navigate academic challenges more effectively. Such approaches are particularly relevant in high pressure higher education contexts, where eliminating stress may be unrealistic, but cultivating students’ psychological resources remains both feasible and educationally actionable.

Several limitations warrant consideration. First, the cross-sectional design does not permit causal inference; future longitudinal and experimental studies are needed to clarify temporal ordering and causal mechanisms linking stress perception, motivation, and well-being. Second, exclusive reliance on self-report measures introduces the possibility of common method variance and social desirability bias. Subsequent research could incorporate multi-method evidence, such as behavioral engagement indicators, academic performance records, learning analytics, or teacher assessments, to strengthen measurement robustness. Third, the partial mediation pattern suggests that additional mechanisms likely operate alongside motivation. Future work should extend the model to include theoretically relevant processes (e.g., coping strategies, emotion regulation, and social support). Physiological or neuroscientific approaches may provide complementary insight into stress processing and regulation, but should be treated as value-added extensions rather than prerequisites for educationally meaningful explanation.

The structural model did not include demographic control variables. Although prior research suggests that gender and academic year may influence stress and motivation, the present study focused on psychological processes rather than group differences. Future studies may incorporate demographic controls to examine potential moderating effects.

Overall, the present study contributes to the growing literature on stress appraisal and student psychological functioning by highlighting the role of academic motivation as a potential psychological pathway linking stress perception and mental well-being. These findings provide insights for educational practitioners aiming to support students’ adaptive engagement under academic pressure. However, findings should be interpreted within the sampled universities and may not generalize to all higher education contexts.

## Data Availability

The datasets presented in this study can be found in online repositories. The names of the repository/repositories and accession number(s) can be found at: https://doi.org/10.6084/m9.figshare.29355341.
